# Duplex Vertical-Flow Rapid Tests for Point-of-Care Detection of Anti-dsDNA and Anti-Nuclear Autoantibodies

**DOI:** 10.3390/bios14020098

**Published:** 2024-02-12

**Authors:** Rongwei Lei, Hufsa Arain, David Wang, Janani Arunachalam, Ramesh Saxena, Chandra Mohan

**Affiliations:** 1 Department Biomedical Engineering, University of Houston, Houston, TX 77204, USA; rlei@uh.edu (R.L.); hufsaarain1@gmail.com (H.A.); jananiarunachalam123@gmail.com (J.A.); 2 John Sealy School of Medicine, UT Medical Branch, Galveston, TX 77555, USA; davwang@utmb.edu; 3 UT Southwestern Medical Center, Dallas, TX 75390, USA; ramesh.saxena@utsouthwestern.edu

**Keywords:** anti-nuclear antibody, anti-dsDNA antibody, vertical-flow assay, gold nanoparticles, rheumatic disease

## Abstract

The goal of this study is to develop a rapid diagnostic test for rheumatic disease and systemic lupus erythematosus (SLE) screening. A novel rapid vertical flow assay (VFA) was engineered and used to assay anti-nuclear (ANA) and anti-dsDNA (αDNA) autoantibodies from systemic lupus erythematosus (SLE) patients and healthy controls (HCs). Observer scores and absolute signal intensities from the VFA were validated via ELISA. The rapid point-of-care VFA test that was engineered demonstrated a limit of detection of 0.5 IU/mL for ANA and αDNA autoantibodies in human plasma with an inter-operator CV of 19% for ANA and 12% for αDNA. Storage stability was verified over a three-month period. When testing anti-dsDNA and ANA levels in SLE and HC serum samples, the duplex VFA revealed 95% sensitivity, 72% specificity and an 84% ROC AUC value in discriminating disease groups, comparable to the gold standard, ELISA. The rapid αDNA/ANA duplex VFA can potentially be used in primary care clinics for evaluating patients or at-risk subjects for rheumatic diseases and for planning follow-up testing. Given its low cost, ease, and rapid turnaround, it can also be used to assess SLE prevalence estimates.

## 1. Introduction

Antinuclear antibodies (ANAs) are a group of autoantibodies that recognize and target nuclear macromolecules and their complexes [1]. Due to its high sensitivity, ANA testing is utilized as screening tool for many autoimmune diseases, such as mixed connective tissue disease, systemic sclerosis, primary Sjogren’s syndrome, autoimmune hepatitis, and systemic lupus erythematosus (SLE) [2,3,4,5,6,7,8,9,10]. The 2019 European League Against Rheumatism (EULAR) and the American College of Rheumatology (ACR) classification of SLE requires an ANA titer of ≥1:80 (Hep-2 Indirect immunofluorescence or equivalent test) as the entry criterion since sensitivity nears 100% [11,12]. Furthermore, while most SLE patients are ANA-positive, ANA-positivity may not be specific for SLE [13].

ANA testing alone is therefore not sufficient for a diagnosis of SLE due to its lack of specificity. In addition to other autoimmune diseases, SLE can be detected in the general population, particularly with increasing age. Approximately 14% of Americans 12 years and older have an ANA titer ≥ 1:80 [14]; studies in other countries estimate the prevalence range to be from 7 to 18% [15,16,17]. On the other hand, the presence of anti-double-stranded DNA antibodies (anti-dsDNA) is highly specific for SLE and is weighted significantly for diagnosis. A positive anti-dsDNA test alone counts as 6 points in the 2019 EULAR/ACR criteria for SLE [11]. Approximately 60–83% of SLE patients have been found to have detectable levels of anti-dsDNA at some point during their illness [18,19,20], and the specificity of anti-dsDNA ranges from 88% to 99% depending on the testing method (acceptable by EULAR/ACR standards) used [21,22,23]. One landmark cohort study found that 55% of SLE patients (*n* = 130) exhibited elevated anti-dsDNA levels up to 9.3 years before diagnosis [24].

There are several methods to detect ANA, most notably the indirect immunofluorescence ANA-IIF and ELISA techniques. The ACR and EULAR currently endorse ANA-IIF for ANA detection [25]. ANAs are assayed using various techniques, with the gold standard being the fluorescent ANA test [26,27]. ANA testing with ELISA was introduced to save time and improve objectivity.

As laboratory-based antibody testing continues to be emphasized in the diagnostic process, point-of-care tests (POCTs) have become increasingly useful for early diagnosis, rapid diagnosis, and diagnostic efficiency. POCTs exhibit convenience, cost-effectiveness, and accurate/reliable results, whether utilized in a laboratory, or a clinical or nonprofessional setting. The benefits extend to both the provider and the patient. Providers save time by obtaining quicker results and can therefore make timely medical decisions [28,29]. A comprehensive review of current and emerging POCTs has recently been published [30]. In many cases, skilled technicians are not even needed, and the performance of the test can be delegated to the patient [31]. This provides patients with the opportunity to play a more active role in their health, providing clinical outcomes that are similar to or better than those obtained under professional care management.

One emerging POCT technology is the vertical flow assay (VFA), which was developed in response to the need for rapid testing and multiplex detection, which the lateral flow assay (LFA) falls short in [32,33,34,35]. As reviewed, the VFA exhibits a faster turnaround time, higher multiplex capability, and higher sample volume capacity characteristics than the LFA does [36]. The LFA utilizes horizontal sample flow on a test strip [37,38,39]. Therefore, its multiplexing capabilities are hindered by the nature of horizontal flow and the physical shape of the test strip. The VFA addresses these issues by utilizing downward flow. Gravity assists in the capillary action of fluids, which helps yield faster results when compared to those obtained under lateral flow [40,41].

Quicker and more convenient detection methods are needed to achieve earlier diagnoses of SLE, as this can have a significant impact on survival and quality of life [42,43]. Anti-dsDNA levels are often used to track the progression and severity of SLE since they correlate closely with the Systemic Lupus Erythematosus Disease Activity Index 2000 (SLEDAI-2K) [44,45,46]. Furthermore, SLE patients who experience significant increases in serum anti-dsDNA upon diagnosis are more likely to have renal disease, and surges in anti-dsDNA titers are predictive of severe lupus flares [47,48,49,50,51]. Utilizing the VFA to rapidly measure ANA and anti-dsDNA levels may thus have a profound impact on the clinical management of SLE. This work is designed to explore this possibility.

## 2. Materials and Methods

### 2.1. Design of the VFA

**Figure 1:**
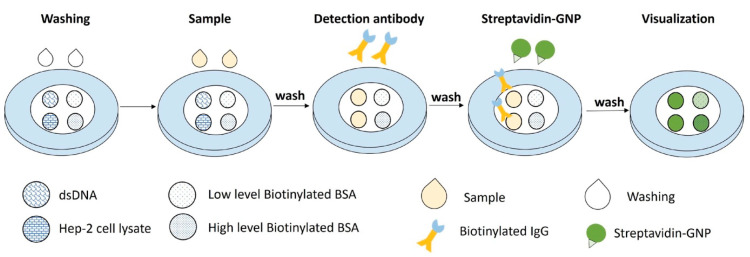
The general principle of the VFA setup. dsDNA and Hep-2 cell nuclear antigens are spotted on the left as test zones, and biotinylated BSA at low and high concentrations are spotted on the right of the nitrocellulose membrane (NCM) as part of control zones. After wetting the NCM, the sample containing anti-dsDNA and/or ANAs is loaded for them to bind to their respective capture antigen substrate on the NCM, thus forming an antigen–antibody immuno-complex. Biotinylated anti-human IgG is then added for it to bind with the anti-dsDNA or ANAs. Finally, streptavidin-conjugated GNPs are added for them to bind with anti-IgG in test zones and with biotinylated BSA in control zones, displaying a green colorimetric signal on the NCM. Between each step, the membrane is washed to remove unbound materials and to prevent nonspecific binding.

The general principle of the VFA setup is as follows: anti-dsDNA and ANA autoantibodies in a sample bind to their respective capture antigen substrate (dsDNA and nuclear antigens) on a nitrocellulose membrane (NCM) and form an antigen–antibody immuno-complex. Biotinylated anti-human IgG is then added to bind with the anti-dsDNA or ANAs. Finally, streptavidin-conjugated gold nanoparticles (GNP) are added to bind with the anti-IgG, displaying a green colorimetric signal on the NCM. Between each step, the membrane is washed to remove unbound materials and to prevent nonspecific binding, as shown in Figure 1.

The rapid vertical flow immunoassay development system and universal buffer were purchased from Cytodiagnostics. Bovine serum albumin (BSA), Tween-20, sodium chloride (NaCl), Tris-HCl, phosphate-buffered saline (PBS, pH 7.4), polyethylene glycol with an average mol wt of 6000 (PEG 6000), and polyvinylpyrrolidone with an average mol wt of 40,000 (PVP40) were purchased from Sigma (St. Louis, MO, USA), diluted in MilliQ water and then filtered using a 0.2 µm filter (Pall Science, New York, NY, USA). dsDNA (Sigma), Hep-2 cell lysate (source of nuclear antigens for ANA testing; Novus Biologicals), Bovine AGE-BSA Biotinylated Protein (R&D system, Minneapolis, MN, USA), human anti-dsDNA standard plasma (708 IU/mL, PSG Lot ID: 20400), biotinylated anti-IgG (Abcam, Eugene, OR, USA), and 150 nm streptavidin-conjugated GNP (Nanocomposix, San Diego, CA, USA) were purchased for VFA development. In addition, a double-stranded DNA (dsDNA) IgG ELISA kit and ANA Screen ELISA kit were purchased from ORIGENE (Rockville, MD, USA) for assay validation. mBSA (methylated bovine serum albumin, Sigma, anti-IgG horseradish peroxidase (Abcam), gelatin (Sigma), ethylenediaminetetraacetic acid (EDTA) (Fischer, TX, USA), and TMB (3,3′,5,5′-Tetramethylbenzidine, Thermo Fisher, Waltham, MA, USA) were purchased for in-house ELISA validation.

### 2.2. Test Samples

Only archived, de-identified samples were used for the study. Prior to adding to the archive, all samples were obtained with written informed consent. Archived de-identified serum samples (19 active LN, 1 inactive SLE, and 2 SLE samples without valid demographics) were obtained from UT Southwestern Medical Center (Dallas, TX, USA). All SLE patients met the 2012 Systemic Lupus International Collaborating Clinics SLE classification criteria. Additionally, active renal SLE (lupus nephritis, LN) activity was evaluated based on a subset of the Systemic Lupus Erythematosus Disease Activity Index (SLEDAI) 2000 criteria, designated as the renal Systemic Lupus Erythematosus Disease Activity Index (rSLEDAI). Any rSLEDAI score > 0 was considered active LN. The healthy controls (HCs) were volunteers with no self-reported conditions. This study was approved by the Institutional Review Boards at UT Southwestern Medical Center and the University of Houston. The demographic information of the serum samples is detailed in Table 1. Serum samples were aliquoted and stored at −20 °C. There was no additional centrifugation before applying samples onto the VFA. 

In addition to the above subjects, one inactive SLE subject was also included in the testing panel. SLEDAI: Systemic Lupus Erythematosus Disease Activity Index; rSLEDAI: renal SLEDAI; LN: lupus nephritis.

### 2.3. Comparison with ELISA

All clinical samples tested via the VFA were also tested using commercial ELISA. Following the manufacturer’s instructions, the plates were transferred to a microplate reader (ELX808, BioTek Instruments, Winooski, VT, USA), and optical density was measured at 450 nm.

### 2.4. Statistical Analysis

The images were first inspected by the naked eye, then captured using an iPhone 12, and then analyzed using Image J. Dose–response curves were generated using Excel 2007, while comparisons between the VFA and commercial ELISA were plotted and analyzed using GraphPad Prism 5 (GraphPad, San Diego, CA, USA). Biomarker group comparisons via the VFA and commercial ELISA were analyzed using the Mann–Whitney U-test as datasets were not normally distributed. One-way ANOVA was used to analyze the LoD and linearity (r^2^) metrics for VFA performance evaluation. ROC curves were plotted with the area under the curve to demonstrate the discriminative power of the biomarker as measured via VFA and ELISA. The limit of detection (LoD) was the lowest concentration that could be detected, denoted by the sum of the mean of the blank (*n* = 2) and three times the standard deviation of the blank.

## 3. Results

### 3.1. Detection of Antinuclear Antibody Using Spiked Human Anti-dsDNA Positive Control Standard and the Duplex VFA

**Figure 2:**
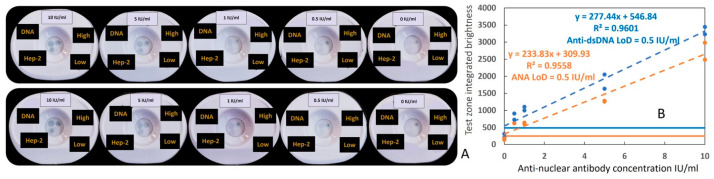
Serial dilution testing using the duplex ANA–αDNA VFA. (**A**) Image of serial dilutions of the positive control standard (ANA+ and αDNA), ranging from 10 IU/mL to 0 IU/mL, applied to the duplex ANA–αDNA VFA in duplicate (top and bottom). (**B**) Dose–response curve created using a human anti-dsDNA positive control standard (ANA+ and αDNA+); plotted is the intensity observed in the ANA test zone (Hep-2, orange dashed line) and αDNA test zone (DNA, blue dashed line) versus the actual concentration applied. Colorimetric intensity values were calculated using ImageJ 1.52a. The LoD was determined to be the lowest assayed concentration, the intensity of which exceeded the threshold of the sum of the mean intensity of the non-spiked buffer (0 IU/mL) and 3× its standard deviation.

A human anti-dsDNA positive control standard, which can bind dsDNA and Hep-2 cell (nuclear antigen) lysates, was used as an internal standard to engineer the rapid vertical flow immunoassay development system to detect anti-dsDNA and ANAs using gold nanoparticles (GNP). To develop the duplex VFA, we first optimized the antigen concentration (Hep-2 cell lysates; dsDNA), biotinylated detection antibody and streptavidin-conjugated GNP concentrations, the clinical sample dilution factor, and buffer components, as shown in Supplementary Figure S1. Later, following assay protocol-1 (Supplementary Method-1), positive samples were prepared by serially diluting a human anti-dsDNA positive control standard (ANA+ and αDNA+), while the assay diluent was used as the negative control. Each test was run in duplicate. As shown in Figure 2A,B, the serial dilution of the positive standard demonstrated a corresponding decrease in αDNA and ANA test zones’ signal intensity, whereas the “High” and “Low” controls zones maintained their respective intensities. For the detection of both antibodies, the duplex assay showed a limit of detection (LoD) of 0.5 IU/mL and a reportable range from 0.5 IU/mL to 10 IU/mL.

### 3.2. Impact of Storage Duration on the Performance of the ANA–αDNA VFA

**Figure 3:**
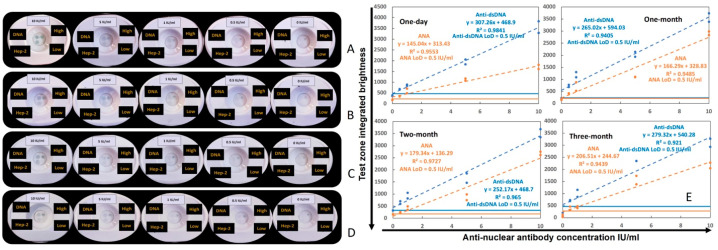
Assessing the storage stability of the duplex ANA–αDNA VFA. Images shown pertain to four sets of standard curves generated using duplex ANA–αDNA VFAs that were stored at room temperature for one day (**A**), one month (**B**), two months (**C**), and three months (**D**) after the immobilization of the antigen on the NCM, in duplicate. Serially diluted human anti-dsDNA positive control standard (ANA+ and αDNA+) from 10 IU/mL, 5 IU/mL, 1 IU/mL, and 0.5 IU/mL to 0 IU/mL were loaded onto the NCM to construct the standard curves. (**E**) Plots of serial dilution of the anti-dsDNA positive control standard (ANA+ and αDNA+) under different storage durations. Orange line indicates ANA and blue line indicates αDNA detection. The LoD of the four sets of standard curves was 0.5 IU/mL for ANA and αDNA detection, and there was no significant difference between the storage times among LoDs and linearities as assessed via one-way ANOVA. The LoD was determined to be the lowest assayed concentration, the intensity of which exceeded the threshold of the sum of the mean intensity of the non-spiked sample (0 IU/mL) and 3× its standard deviation.

The stability of the duplex VFA was evaluated per assay protocol-1 (Supplementary Method-1) through storage in a desiccator at room temperature over three months. After one-day, one-month, two-month, and three-month storage, the same standard curve as above was run by serially diluting the human anti-dsDNA positive control standard (ANA+ and αDNA+). In Figure 3A–D, the images of four sets of standard curves are shown in order of increasing storage time. The linearity and LoD of the four sets of serial dilutions are shown in Figure 3E. A one-way ANOVA nonparametric test was used to compare the linearity and LoD of the four sets of data, and no significant differences were found between the storage conditions, indicating that storage time had no impact on VFA performance.

### 3.3. Inter-Operator Coefficient of Variation in Operating the Duplex ANA–αDNA VFA

To verify the reproducibility of running and reading the duplex ANA–αDNA VFA, two operators ran the same serial dilution using the ANA–αDNA VFA, and the inter-operator coefficient of variation (CV) was calculated for each concentration point, as shown in Table 2. The average inter-operator CV was 12.3% for reading anti-dsDNA levels and 19% for reading ANA levels.

### 3.4. Testing Clinical Serum Samples Using the Duplex ANA–αDNA VFA and ELISA

To facilitate the running of clinical samples, all-purpose GNP diluent-2, which can be used for diluting serum samples and for detecting Ab and GNP (Figure S2), was used. Serum autoantibody levels in 22 SLE and 18 HC serum samples were assayed using the duplex ANA–αDNA VFA using assay protocol-2 (Supplementary Method-2); images are shown in Figure S3. The observer score (OS) of each assay was recorded based on Supporting Method-3 (Figure S4) by three researchers through visual intensity scores, which are shown in Supplementary Figure S5.

**Figure 4:**
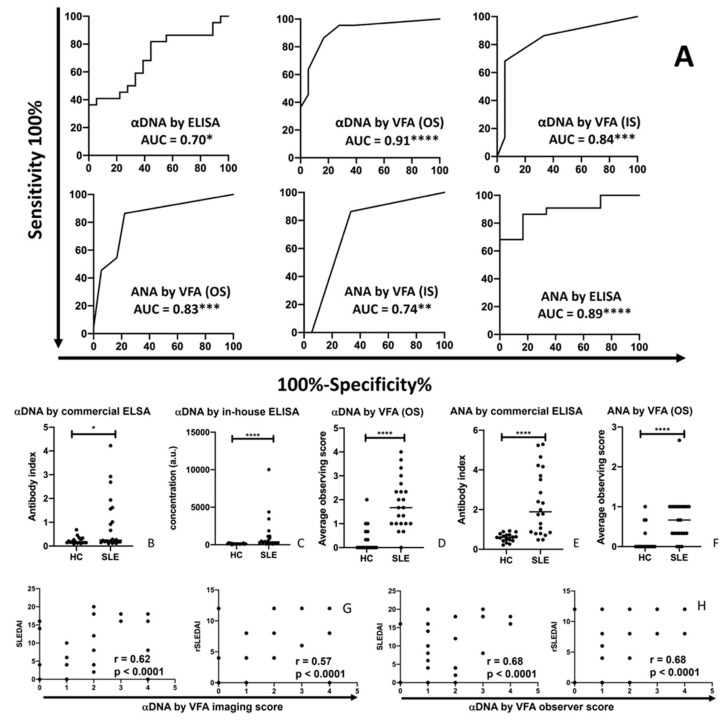
Disease-discriminating power of the duplex ANA–αDNA VFA and ELISA. (**A**) Serum autoantibody levels in 22 SLE and 18 HC serum samples assayed using the duplex ANA–αDNA VFA (OS or IS) and ELISA. Their ability to discriminate SLE patients from HCs was assessed using ROC AUC values. (**B**–**F**) The levels of autoantibodies assayed in SLE patients and HCs using the 2 different platforms plotted and analyzed using a Mann–Whitney U-test. (**G**,**H**) SLEDAI and rSLEDAI disease activity values (*Y*-axis) in all SLE patients with these values (20 out of 22) plotted against αDNA levels (*X*-axis), as assayed via the VFA (IS or OS), with Pearson’s correlation. * *p* < 0.05, ** *p* < 0.01, *** *p* < 0.001, **** *p* < 0.0001 as determined using Mann–Whitney U-test. AUC: area under the curve; IS: imaging score; OS: observer score; SLEDAI: Systemic Lupus Erythematosus Disease Activity Index; rSLEDAI: renal SLEDAI.

The same serum samples from 22 SLE patients and 18 HCs tested via the VFA were also evaluated using commercial ELISA assays for anti-dsDNA and ANA detection. The OS and IS of the duplex ANA–αDNA VFA showed good discriminating power for distinguishing SLE patients from HCs, as did the commercial ELISA, based on ROC AUC analysis, as shown in Figure 4A. The αDNA levels in SLE patients were significantly higher than those in HCs when assayed via ELISA (*p* < 0.05, Figure 4B,C), or via the VFA (*p* < 0.0001, Figure 4D). ANAs in SLE patients were also significantly higher than those in HCs when assayed via ELISA (*p* < 0.0001, Figure 4E), which is in good agreement with the measurement of ANA via the VFA (*p* < 0.0001, Figure 4F). Similar findings were observed when we used an in-house αDNA ELISA, as shown. 

The SLEDAI and rSLEDAI indices in the 22 SLE patients (19 active LN patients, 1 inactive SLE patient, and 2 patients without a valid SLEDAI or rSLEDAI value) exhibited significant Pearson’s correlations with serum αDNA levels, as assayed via the VFA (OS or IS), with correlation coefficients typically exceeding 0.6 (Figure 4G,H), alluding to their potential clinical utility.

## 4. Discussion

There is clearly an unmet need for identifying improved biomarkers for SLE and LN, as suggested elsewhere [52]. In addition, it is also imperative to design better ways to detect these biomarkers and to enable patients to self-monitor their disease from the comfort of their home. Technologies that allow for the point-of-care (POC) monitoring of disease are certainly needed. One such approach is a recently described LFA method for monitoring urinary disease biomarker ALCAM and urine normalizer HVEM [39], in contrast to conventional ELISA for the biomarker’s detection [53,54]. As opposed to POC devices for detecting protein biomarkers, there are currently no reports of POC devices for monitoring autoantibodies in SLE/LN. Given that anti-dsDNA and ANA autoantibodies are highly associated with lupus [11,12,18,19,20,21,22,23,24], and can even be predictive of disease activity in SLE/LN [44,45,46,47,48,49,50,51,55], it is important to engineer POC approaches to make this feasible. The reported work meets this unmet need.

Two POC technologies were considered, namely the LFA and the VFA. The latter was selected in view of its several advantages, including its faster turnaround time, higher multiplex capability, and potentially higher sensitivity [36]. Furthermore, we proceeded to design a duplex VFA capable of detecting two of the most important diagnostic autoantibodies in SLE (anti-dsDNA and ANA) within a single assay kit. The engineered duplex VFA displayed minimal background signals for the negative control and brightly colored positive zones varying linearly with serum antibody concentrations. The duplex VFA showed a limit of detection of 0.5 IU/mL for both autoantibody targets and demonstrated good inter-operator reproducibility as well as storage stability up to the maximum period tested, 3 months. With actual clinical samples, the engineered duplex ANA–αDNA VFA displayed a diagnostic power of 0.84 according to the observer score in distinguishing SLE sera from HC sera, comparable with the gold standard, ELISA. Moreover, the ANA and anti-dsDNA levels measured using the engineered VFA correlated significantly with both disease activity indices, the SLEDAI and renal SLEDAI. Of note, the VFA enabled the duplex detection of both ANA and αDNA within 10 min, in contrast to the gold standard ELISA, which is single-plex and requires several days for completion in clinical laboratories. Moreover, an ELISA assay can only be performed in a laboratory setting, using equipment such as readers and washers, by a trained technician. In contrast the 10 min turnaround time of the duplex VFA enables POC testing in primary-care clinics and even home testing.

This duplex VFA can potentially be used in primary-care clinics for the screening of patients where rheumatic diseases are considered a differential diagnosis. Since a large fraction of autoimmune rheumatic diseases are documented to be ANA-positive, this point-of-care test (which can be completed in 10 min) will enable the clinician to triage patients for confirmatory autoantibody panel testing or for referrals to a rheumatologist. On the other hand, new patients and/or high-risk subjects (e.g., first-degree relatives of SLE patients) who test positive for ANA and anti-dsDNA may be selected for confirmatory tests to confirm the diagnosis of SLE. The transition from ANA seropositivity to anti-DNA seropositivity can also be captured using this duplex VFA, particularly in pre-SLE or pre-clinical SLE patients. Moreover, the correlation of the assayed antibodies with the SLEDAI and rSLEDAI also suggests that this duplex VFA may enable the routine monitoring of disease activity in patients with SLE. In fact, regular home-based monitoring is also feasible using either a drop of blood or saliva, as we have reported elsewhere [56,57].

Several aspects of this study and the VFA design could be enhanced. The sample size could be expanded even further to include a larger number of participants, drawn from established cohorts. The engineered duplex VFA serves as an illustration of point-of-care (POC) testing utilizing serum samples. For scenarios in which a centrifuge is unavailable, the assay can be integrated with the application of Cleanascite, a reagent designed to selectively remove lipids, fats, impurities, and other cellular debris from samples. Alternatively, the Folch and Bligh–Dyer methods can be employed as substitutes. Nevertheless, a notable advantage of the VFA lies in its improved multiplexing capability. As new disease diagnostic biomarkers emerge in the forthcoming years, this established protocol and point-of-care testing approach can be readily extended to encompass these additional markers.

## 5. Conclusions

Methods commonly utilized to detect SLE-associated antibodies such as immunofluorescence or ELISA are time-consuming and resource-intensive. The engineered duplex VFA test reported here was formulated based on the sandwich immunoassay principle. Here, we engineered a highly specific vertical flow-based point-of-care test for the rapid screening of rheumatic disease and SLE. To the best of our knowledge, no published work exists on the rapid detection of ANA and αDNA using a VFA. In this study, the duplex ANA–αDNA VFA demonstrated substantial concordance with conventional ELISA in terms of distinguishing SLE, and exhibited strong agreement with the SLEDAI and rSLEDAI in terms of diagnosing SLE activity. Thus, this point-of-care test holds promise for effectively testing or screening a large number of subjects rapidly and delivering more precise estimates of ANA and/or SLE prevalence, particularly in resource-limited settings.

## Figures and Tables

**Table 1:** Demographics of serum samples used for the VFA.

Variable	Healthy Controls	Active LN
	*n* = 18	*n* = 19
Race		
White	10	2
Black	6	8
Hispanic	0	8
Asian	2	1
Age (yr)		
Mean	31 ± 9.2	30 ± 10.3
Range	18–53	19–60
Sex		
Female	11	12
Male	7	7
SLEDAI		
Mean	N/A	11 ± 6.0
Range	N/A	4–20
rSLEDAI		
Mean	N/A	7 ± 3.5
Range	N/A	412

**Table 2:** Inter-operator coefficient of variation for running the duplex VFA. Inter-operator coefficient of variation in test zone brightness for a single-point concentration, and average inter-operator CV from two operators’ standard curves for reading the test zone brightness using the ANA–αDNA duplex VFA. The tabulated numbers represent the IS, as assessed by ImageJ 1.52a.

IU/mL	ANA		Anti-dsDNA			
	Operator-1	Operator-2	CV%	Operator-1	Operator-2	CV%
10	1711	2735	33	3561	3338	5
5	1121	1271	9	1944	1841	4
1	598	622	3	860	1054	14
0.5	349	624	40	676	818	13
0	182	157	11	374	261	25
			19			12

## Data Availability

Data are available upon reasonable request.

## Supplementary Materials

The following supporting information can be downloaded at https://www.mdpi.com/article/10.3390/bios14020098/s1: Figure S1: Optimization of protocols for αDNA VFA and ANA VFA; Figure S2: Optimization of duplex ANA–αDNA VFA for clinical samples; Figure S3: Images of 40 clinical serum samples (22 SLE and 18 HC) tested using the duplex ANA–αDNA VFA; Figure S4: ANA–αDNA VFA reference and test zone semiquantitative reporting; Figure S5: Observer score and imaging score of 40 clinical serum samples tested using the ANA–αDNA VFA.
