# Supplementary material for: Duplex Vertical-Flow Rapid Tests for Point-of-Care Detection of Anti-dsDNA and Anti-Nuclear Autoantibodies
Source: Biosensors (Basel). 2024 Feb 12;14(2):98. doi: 10.3390/bios14020098 (PMC10887294; doi:10.3390/bios14020098)
Supplement: Supplementary file 1 [file biosensors-14-00098-s001.zip › biosensors-2848460-supplementary.pdf]

Supplementary methods:

1. Performance of the duplex ANA–αDNA VFA using the human anti-dsDNA positive control standard (ANA+, αDNA+)

Assay protocol-1: Since the engineered VFAs will be used to assay two different autoantibodies, these VFAs will be referred to as duplex ANA–αDNA VFAs. An amount of 1 µl of 1 mg/ml of dsDNA in PBS was spotted on the top left of the nitrocellulose membrane (NCM), and this corner is referred to as the αDNA test zone. Briefly, 1 µl of 10 µg/ml of Hep-2 cell lysates (source of nuclear antigens for ANA testing) in PBS was spotted on the lower left of the NCM, and this corner is referred to as the ANA test zone. After air-blowing the NCM for 5 min, the VFA was transferred to a UV lamp for 45 min irradiation. Then, 1 µl of 250 µg/ml of biotinylated BSA in PBS was spotted on the top right of the NCM, and this corner is referred to as the positive control ("High"). An mount of 1 µl of the assay diluent (1% BSA, 0.01% tween 20 in 10 mM PBS) was spotted on the lower right of the NCM, and this corner is referred to as the negative control ("Low"). After air-blowing the NCM for 30 min, the VFA was stored at room temperature with desiccator packets for future use. Initially, three drops of Universal buffer were added to the NCM in the center of the cartridge (approximately 200 µl). Then, 200 µl of the serially diluted human anti-dsDNA/ANA positive control antibody standard (ANA+, αDNA+) in the assay diluent was loaded onto the membrane, followed by three drops of the universal buffer being spotted. Next, 10 µl of 10 µg/ml biotinylated anti-IgG (detection antibody or det-Ab) in the assay diluent was loaded onto the αDNA test zone, ANA test zone, positive control zone, and negative control zone separately, followed by three drops of universal buffer being added. Next, 5 µl of OD=2 streptavidin-conjugated GNP in GNP diluent-1 (20 mM Tris, 150 mM NaCl, 1% BSA, 0.1% Tween 20, pH=8, filtered) was loaded onto the αDNA test zone, ANA test zone, and "High" control and "Low" control zones separately. Finally, ten drops of universal buffer were loaded, as a wash step.

2. Evaluation of the duplex ANA–αDNA VFA using clinical samples

Assay protocol-2: On the top and lower left side of the NCM, 0.7 µl of 50 µg/ml of dsDNA and 5 µg/ml of Hep-2 cell lysate in PBS were spotted as αDNA and ANA test zones, respectively. "Low" and "High" control zones were introduced to serve as a cut-offs for reporting semiquantitative results. At the top right and lower right of the NCM, 0.7 µl of 50 µg/ml of biotinylated BSA and 800 µg/ml of biotinylated BSA in GNP diluent-2 (20 mM Tris, 150 mM NaCl, 0.2% Tween 20, 1% BSA, pH=8, filtered) were spotted, respectively, to serve as "Low" and "High" control zones. GNP diluent-2 was used as an all-purpose dilution buffer for clinical samples, det-Ab, and GNP preparation, while universal buffer was used only for washing. A 1/100 serum dilution was used for all clinical samples as this has been optimal in other assay platforms. First, three drops of universal buffer were placed on the NCM, and then 200 µl of 100-fold diluted serum sample in GNP diluent-2 was loaded to the whole NCM, followed by three drops of the universal buffer being added. Next, 10 µl of 5 µg/ml det-Ab in GNP-2 diluent was loaded onto the NCM αDNA test zone and ANA test zone followed by three universal buffer drops being added. Next, 5 µl of OD = 2 streptavidin-conjugated GNP in GNP diluent-2 was individually loaded onto the ANA and αDNA test zones, and 2 µl of OD=2 GNP in GNP diluent-2 was loaded individually onto the "High" and "Low" control zones. Finally, seven drops of universal buffer were loaded as a wash step.

3. Semiquantitative recording of duplex ANA-αDNA VFA

ANA and αDNA test zone results were recorded using an "Observer Score" (OS) from 0 (no visible trace or significantly less visible signal than "Low"), 1+ (similar to the intensity of "Low"), 2+ (higher than

“Low” but lower than “High”), and 3+ (similar to the intensity of “High”) to 4+ (higher than “High”) based on how the signal appears to the naked human eye, shown in Figure S4. Additionally, the intensity of each VFA spot was quantified using ImageJ and recorded as an “Imaging score” (IS). In order to convert the IS into a semi-quantitative score that could be used across all VFAs, each IS was first expressed as a range, from (0.81 * IS) to (1.19 * IS). For example 3000 IS units on the test zone were read as 2430 to 3570 IS units. For the semi-quantitative reporting of this readout, the IS intensity was be recorded as 0 if the upper limit of the test zone IS score was less than the “Low” control zone IS intensity. It was recorded as 1+ if the lower limit of the test zone IS score was lower than the “Low” control zone intensity but the upper limit of the test zone IS score was higher than the “Low” control zone intensity. It was be recorded as 2+ if the lower limit of the test zone IS score was higher than the “Low” control zone intensity but the upper limit of the test zone IS score was less than the “High” control zone intensity. It was be recorded as 3+ if the upper limit of the test zone IS score was greater than the “High” control zone intensity but the lower limit fell below this. Finally, it was be recorded as 4+ if the lower limit of the test zone IS score was greater than the “High” control zone intensity.

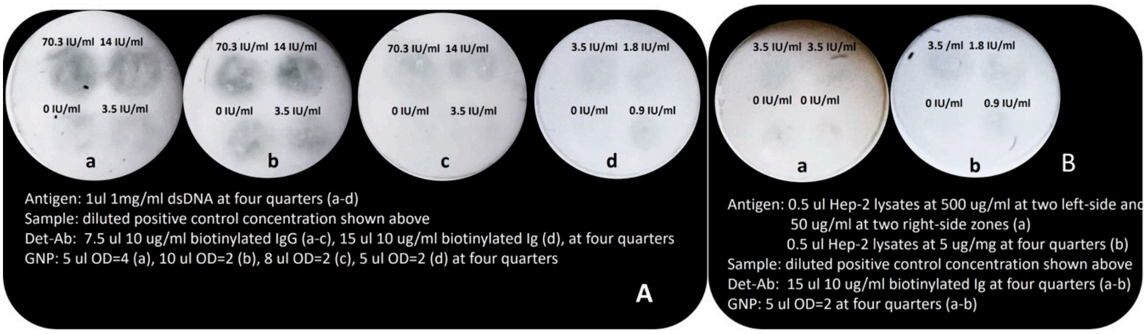

Figure S1. Optimization of protocols for αDNA VFA and ANA VFA. A) An amount of 0.5 μl of 1 mg/ml of the dsDNA antigen immobilized onto the four quarters of the NCM of the αDNA VFA (a-d). The positive control samples (spiked assay diluent) and negative control samples (non-spiked assay diluent) were prepared and loaded onto the four quarters of the NCM in the concentrations indicated in the figure (a-d). Using 7.5 μl of 10 μg/ml of det-Ab in all quarters of the NCM (a-c), 5 μl of OD=4 GNP (a), 10 μl of OD=2 GNP (b), and 8 μl of OD=2 GNP (c) were loaded onto the four quarters of the αDNA VFA NCM, individually. An amount of 15 μl of 10 μg/ml of det-Ab in all four quarters of the NCM was used (d) with 5 μl of OD=2 GNP on the four quarters of the NCM on the αDNA VFA. **B-a)** Briefly, 0.5 μl of 500 μl/ml and 50 μg/ml of Hep-2 cell lysate were spotted in the two left-side zones and two right-side zones of the NCM of the ANA VFA. The positive control samples (assay diluent spiked with standards) and negative control samples (non-spiked assay diluent) were prepared and loaded onto the four quarters of the NCM in the concentrations indicated on the figure. Under the test condition of 15 μl of 10 μg/ml of det-Ab and 5 μl of OD=2 GNP added to the four quarters, the membrane showed the background in the negative zone (0 IU/ml). **B-b)** Briefly, 0.5 μl of 5 μg/ml of Hep-2 cell lysate were spotted on the four quarters of the ANA VFA NCM. Under the test condition of 15 μl of 10 μg/ml of det-Ab and 5 μl of OD=2 GNP, the negative zone (0 IU/ml) remained clean. NCM: nitrocellulose membrane; det-Ab: biotinylated IgG; GNP: streptavidin-conjugated gold nanoparticles.

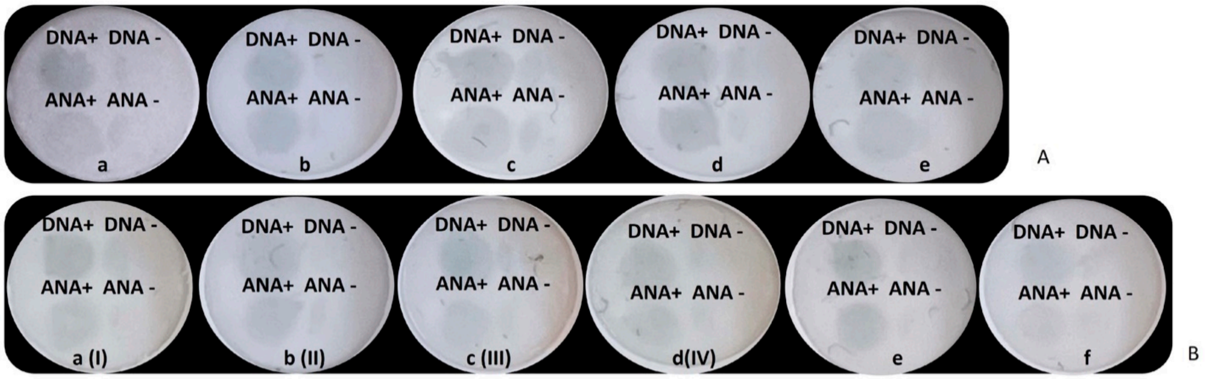

Figure S2. Optimization of duplex ANA–aDNA VFA for clinical samples.
A (a-c) Optimization of antigen concentration and detection antibody. In total, 100 μg/ml dsDNA and10 μg/ml of Hep-2 cell lysates (a), 100 μg/ml dsDNA and 5 μg/ml of Hep-2 cell lysates (b), and 50 μg/ml dsDNA, and 5 μg/ml of Hep-2 cell lysate (c) were evaluated using a 200-fold diluted positive patient sample (+) and a healthy negative control sample (-). **A (d-e)** Using 50 μg/ml dsDNA and 5 μg/ml Hep-2 cell lysate antigen concentrations (d, e), 10 μl of 10 μg/ml of det-Ab (d) and 10 μl of 5 μg/ml of det-Ab (e) were evaluated using the same positive (+) and negative samples (-) as above. **B (a-d)** Evaluation of four types of GNP diluents. I: 20 mM Tris, 150 mM NaCl, 1% BSA, 0.1% Tween 20; II: 20 mM Tris, 150 mM NaCl, 1% BSA, 0.2% Tween 20; III: 20 mM Tris, 150 mM NaCl, 1% BSA, 0.1% Tween 20, 0.5% PVP40; and IV: 20 mM Tris, 150 mM NaCl, 1% BSA, 0.1% Tween 20, and 0.5% PEG, all at pH 8, filtered. Briefly, 50 μg/ml dsDNA and 5 μg/ml Hep-2 cell lysate antigen concentrations are the same as those of the positive (+) and negative samples (-) above; 10 μl of 10 μg/ml det-Ab and 5 μl of OD=2 GNP were prepared in the corresponding GNP diluents, I-IV. GNP diluent-II showed more homogenous dispersion of GNP and better distinction between positive (+) and negative (-) serum. **B (e-f)** Using 50 μg/ml dsDNA and 5 μg/ml Hep-2 cell lysate antigen concentrations and the GNP diluent-II buffer system, 10 μl of 5 μg/ml and 5 μl of 5 μg/ml of det-Ab were further evaluated in the duplex VFA, revealing 10 μl of 5 μg/ml det-Ab as able to yield an optimal signal to noise ratio. Det-Ab: det-Ab: biotinylated IgG; GNP: streptavidin-conjugated gold nanoparticles.

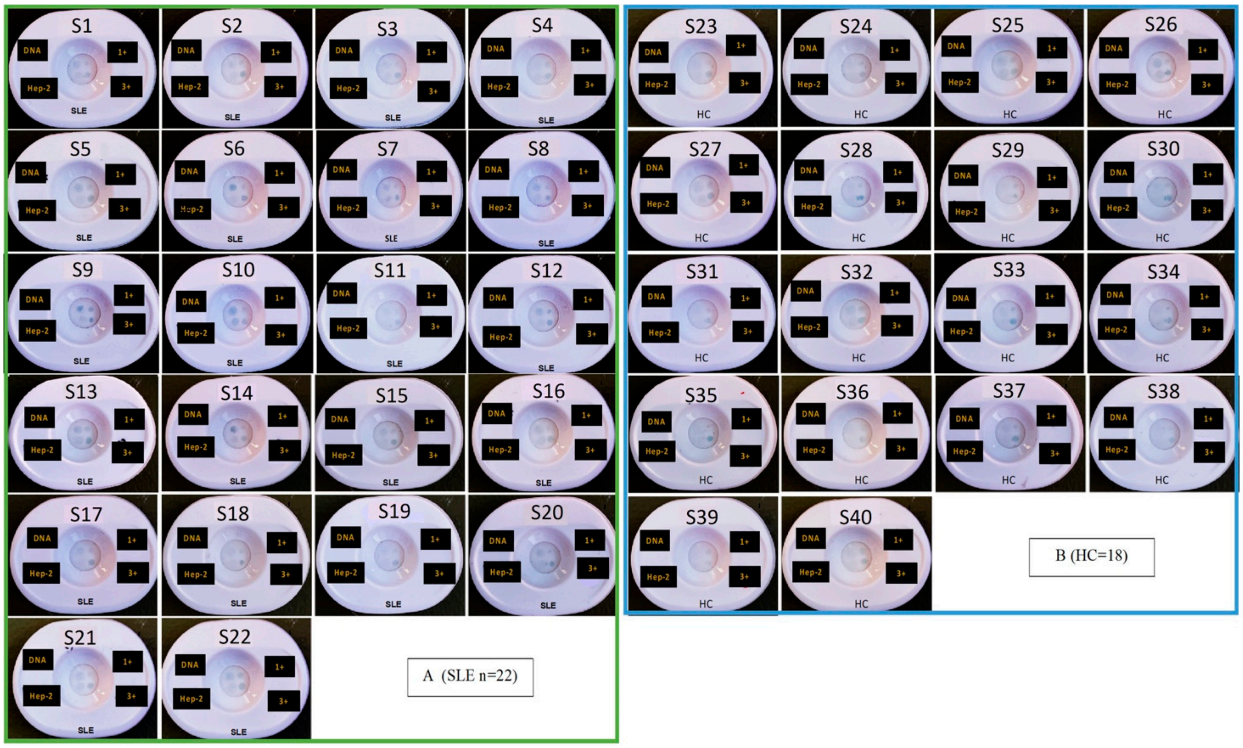

Figure S3. Images of 40 clinical serum samples (22 SLE and 18 HC samples) tested using the duplex ANA–aDNA VFA. Following assay protocol-2, at the top and lower left of the NCM, 0.7 µl of 50 µg/ml of dsDNA and 5 µg/ml of Hep-2 cell lysate in PBS were spotted as αDNA (DNA) and ANA test zones (Hep-2), respectively. At the top right and lower right of the NCM, 0.7 µl of 50 µg/ml of biotinylated BSA and 800 µg/ml of biotinylated BSA in G NP diluent-2 were spotted to serve as "1+" and "3+" control zones. GNP diluent-2 was used for the dilution of clinical samples, det-Ab, and GNP, while universal buffer was used only for washing. First, three drops of universal buffer were placed on the NCM, and then 200 µl of the 100-fold-diluted serum sample was loaded onto the whole NCM, followed by three drops of the universal buffer being added. Next, 10 µl of 5 µg/ml det-Ab was loaded onto the NCM αDNA test zone and ANA test zone followed by three universal buffer drops being added. Next, 5 µl of OD = 2 streptavidin-conjugated GNP was individually loaded onto the ANA and αDNA test zones, and 2 µl of OD=2 GNP was loaded individually onto the "3+" and "1+" control zones. Finally, seven drops of universal buffer were loaded as a wash step. SLE: systemic lupus erythematosus; HC: healthy control.

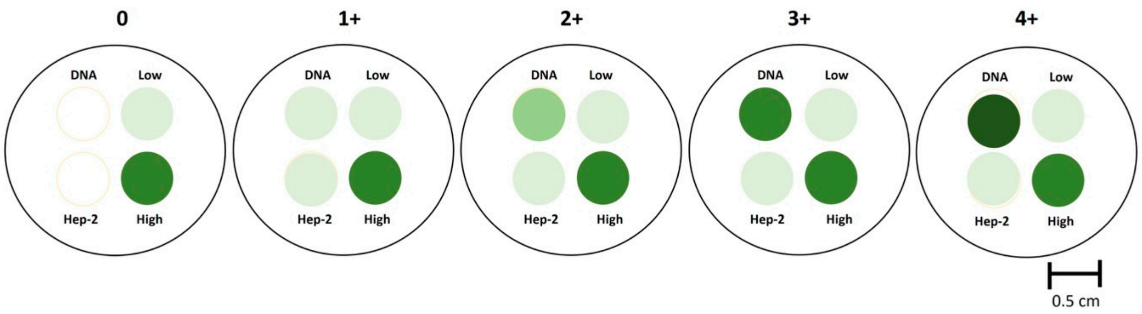

Figure S4. **ANA–αDNA VFA reference and test zone semiquantitative reporting**. The images of the ANA–αDNA VFA demonstrate two test zones indicative of anti-dsDNA and ANA detection and two reference zones used for the semiquantitative reporting of antibody levels. Observer scores (OS) from 0 to 4+ are indicated at the top of each image to show the level of intensities seen in the αDNA test zone (DNA) relative to "Low" and "High" control zone intensities whereas the ANA test zone (Hep-2) shows an OS of 0 in the first image to the left and maintains an intensity of 1+ for the rest of the images, relative to the "Low" and "High" control zone intensities. Thus, whereas the "1+" signal straddles the "Low" calibrator, the "3+" signal straddles the "High" calibrator. The terms "quarters" and "corners" are used interchangeably to refer to the 4 assay zones shown.

ID	OS-1-ANA	OS-1- aDNA	OS-2-ANA	OS-2- aDNA	OS-3-ANA	OS-3- aDNA	OS-Readout	IS-ANA	IS-aDNA	IS-Readout
A30	1	3	1	3	1	2	ANA+/αDNA+	1	2	ANA+/αDNA+
A33	0	1	0	1	1	1	ANA-/αDNA+	1	2	ANA+/αDNA+
A31	1	0	1	1	1	1	ANA+/αDNA+	0	0	ANA-/αDNA-
A36	0	0	0	0	0	0	ANA-/αDNA-	0	0	ANA-/αDNA-
A37	0	2	0	2	1	3	ANA-/αDNA+	1	3	ANA+/αDNA+
A38	0	4	0	4	1	3	ANA-/αDNA+	1	4	ANA+/αDNA+
A39	1	2	1	2	1	2	ANA+/αDNA+	1	2	ANA+/αDNA+
A42	1	2	1	2	1	3	ANA+/αDNA+	1	2	ANA+/αDNA+
A43	0	3	0	3	1	3	ANA-/αDNA+	1	3	ANA+/αDNA+
A44	3	3	2	3	3	4	ANA+/αDNA+	1	4	ANA+/αDNA+
A45	0	1	0	1	0	1	ANA-/αDNA+	1	1	ANA+/αDNA+
A46	0	1	0	1	1	1	ANA-/αDNA+	1	3	ANA+/αDNA+
A50	1	2	1	2	1	3	ANA+/αDNA+	1	2	ANA+/αDNA+
A51	1	4	1	4	1	4	ANA+/αDNA+	1	4	ANA+/αDNA+
A52	0	1	1	1	1	1	ANA+/αDNA+	1	2	ANA+/αDNA+
A54	1	2	1	2	1	1	ANA+/αDNA+	1	2	ANA+/αDNA+
A55	1	2	0	1	1	1	ANA+/αDNA+	1	1	ANA+/αDNA+
B22	0	1	0	1	0	1	ANA-/αDNA+	1	1	ANA+/αDNA+
B26	0	1	0	0	1	1	ANA-/αDNA+	0	0	ANA-/αDNA+
B84	1	2	1	2	1	2	ANA+/αDNA+	1	2	ANA+/αDNA+
B94	1	1	1	2	1	2	ANA+/αDNA+	1	2	ANA+/αDNA+
B95	0	2	0	1	1	1	ANA-/αDNA+	1	1	ANA+/αDNA+
B0050	0	1	0	0	0	0	ANA-/αDNA-	1	1	ANA-/αDNA-
B0051	0	0	0	0	0	0	ANA-/αDNA-	0	0	ANA-/αDNA-
B0052	1	1	0	0	1	2	ANA+/αDNA+	1	1	ANA+/αDNA+
B0053	1	2	1	2	1	2	ANA+/αDNA+	4	4	ANA+/αDNA+
B0054	1	1	0	0	1	2	ANA+/αDNA+	1	1	ANA+/αDNA+
B0055	0	0	0	0	0	1	ANA-/αDNA-	0	0	ANA-/αDNA-
B0056	0	0	0	0	0	0	ANA-/αDNA-	0	0	ANA-/αDNA-
B0058	0	0	0	0	0	0	ANA-/αDNA-	0	0	ANA-/αDNA-
B0059	0	0	0	0	0	0	ANA-/αDNA-	0	0	ANA-/αDNA-
B0061	0	1	0	0	1	1	ANA-/αDNA+	1	1	ANA+/αDNA+
B0063	0	0	0	0	0	0	ANA-/αDNA-	0	0	ANA-/αDNA-
B0064	0	0	0	0	0	0	ANA-/αDNA-	0	0	ANA-/αDNA-
B0065	0	0	0	0	0	0	ANA-/αDNA-	0	0	ANA-/αDNA-
B0079	0	0	0	0	0	0	ANA-/αDNA-	1	1	ANA+/αDNA+
B0111	0	1	0	0	0	1	ANA-/αDNA+	0	0	ANA-/αDNA-
B0113	0	0	0	0	0	0	ANA-/αDNA-	0	0	ANA-/αDNA-
B0110	0	0	0	0	0	0	ANA-/αDNA-	0	0	ANA-/αDNA-
B0112	0	0	0	0	0	0	ANA-/αDNA-	0	0	ANA-/αDNA-

Figure S5. Observer score and imaging score of 40 clinical serum samples tested using the ANA–aDNA VFA. The OS was recorded by three researchers based on the visual intensity shown in Supplementary Figure S3. IS: imaging score; OS: observer score; ANA: anti-nuclear antibody.
